# Supplementary material for: Unpacking polarization: Antagonism and alignment in signed networks of online interaction
Source: PNAS Nexus. 2024 Jul 13;3(12):pgae276. doi: 10.1093/pnasnexus/pgae276 (PMC11655294; doi:10.1093/pnasnexus/pgae276)
Supplement: pgae276_Supplementary_Data [file pgae276_supplementary_data.pdf]

Supporting Information for

Unpacking Polarization: Antagonism and Alignment in Signed Networks of Online Interaction

Emma Fraxanet, Max Pellert, Simon Schweighofer, Vicenç Gómez, David Garcia

Corresponding author: Emma Fraxanet.
E-mail: emmafraxanet@gmail.com

This PDF file includes:

Supporting text
Figs. S1 to S11
Tables S1 to S4
SI References

Supporting Information Text

1. Materials

Figure S1 shows an example of the discussion forums interface of DerStandard. Table S1 shows the temporal and size features of the datasets as well as the final size of the signed networks of relations.

2. Methods

A. Assessment of approximated results. To assess the robustness of the results, given that we use an approximated measure for some of the datasets, we run the partitioning algorithm 200 times for each network and keep the lowest value of frustrated edges and its respective partition. We provide three robustness checks: (i) To assess the number of iterations of the algorithm, we check if we can find the optimal solution within $it = \frac{1}{2}200$ iterations, and if not we see how different would the final result change. We find that the number of frustrated edges of the optimal solution found in half of the iterations differs less than 1% with the one found in 200 iterations. (ii) We compare the similarities between the partitions within the 3 best solutions found: in the three cases we find almost-identical sizes for the obtained groups, with a Szymkiewicz–Simpson overlap coefficient of 0.79. (iii) We provide a comparison analysis between the exact solutions and approximated solutions when using a rolling window for the BW1 dataset, see Figure S2. The groups in the exact and approximated partitions have a Szymkiewicz–Simpson overlap coefficient of 0.8. The Pearson correlation coefficient between the obtained time series is 0.868.

Tables S2 and S3 show the detailed resulting partitions from the exact or approximated methods.

B. Multipartition study. In Figure S3 we show the distribution of the partitioning algorithm results for the approximated method. This method is of stochastic nature and therefore we run it several times (i.e. 200 instances) and select the partition that yields the minimum number of frustrated edges. We also use these results to select the optimal number of groups, k^*, by selecting k with the lowest value of frustrated edges. We can also see how the trend of results seems to increase with k, in agreement with the theorem in (1) which states that the number of minimum frustrated edges is concave when plotted against k. We show the results for the Destandard dataset and for the BW2 dataset, which are the datasets that require the use of the approximated method because of their dimensions.

C. Metrics normalization. In Fig S4 we show the metrics of Divisiveness and Cohesiveness before normalization for the time series of BW1. We show both the original data metrics and the null model mean. This figure supports the normalization choice of subtracting Antagonism (i.e. proportion of negative interactions) from Divisiveness to obtain a more meaningful signal on the relevance of sign distribution in a specific time window. Cohesiveness, on the other hand, is perfectly correlated with the proportion of positive interactions and thus should be normalized by subtracting such amount.

3. Results

A. Antagonism and Alignment in BW1. In Fig S5 we show the metrics of Antagonism and Alignment for the time series of BW1. The two time series have a correlation of 0.616. We find this number to be low enough to identify both metrics as different phenomena and thus to emphasize the importance of considering them separately. Moreover, due to the use of a rolling window for the construction of the time series, this correlation measure also contains auto-correlations, and would otherwise be lower.

B. Size effects. In Figure S6 we show the correlation between our Alignment measures for sub-sets of the data and the size of votes (in the case of a temporal rolling window) or votes, posts and articles (in the case of a topic). These coefficients are computed on the data used for the main text figures. As expected, we see there is no direct correlation between the amount of data we consider for each sub-set and the level of Alignment in the network of interactions.

C. Birdwatch wordshift graphs. We apply a word shift method in order to contextualize the topics of discussion surrounding the peaks we detect in the timeline obtained for BW1. Word shifts extract which words contribute to a difference between two texts and how they do so. We use the tool *Shifterator* (2), which shows the differences in interpretable horizontal bars that compare two texts. Particularly, we use a Frequency-based proportion shift method, that consists on measuring the difference of relative frequencies of a word in each text. In Figures S7, S8 and S9 we show such wordshift graphs for each peak. In Table S4 we show the relevant keywords and their corresponding events found through a news articles search with given keywords and time frames.

D. Derstandard Contextualization: 2015-2016 changes in Austria. It's striking that between 2015 and 2016 a pronounced increase occurs in our Alignment metric for political topics in DerStandard. During that time, media discourse was dominated by the events in Cologne (and other German cities) during the New Year's Eve 15/16 celebrations. A substantial number of women was reporting sexual assault by groups in public, an unusual criminal offense in Germany (for a timeline of events and contextualisation see (3) and Wikipedia page). The political discussion starting with those events led to a pronounced shift in public opinion, summarized by the influential German newspaper "Der Spiegel" as such: "The night brought an end to the sense of euphoria that had accompanied the welcoming of hundreds of thousands of refugees into the country earlier that year".

4. Discussion

A. Using frustrated edges as a metric of polarization (alignment). In our work, we understand frustration as the number of frustrated edges given a partition (whether it is optimal for that set of edges or not). Frustration has been previously used to define partial balance (4). However, that is not what we intend to measure in our work. Alignment, the index we define, has another goal even if inspired by a partial balance index: to measure how well a network of interactions fits into a previously determined partition. In other words: how aligned the votes users give to each other are to the main fault line.

This metric is informative of how much users are coherent with the faction to which they belong, which inherently is related to polarization. We also mention in the section "Partitioning methods for signed networks", under "Previously defined useful metrics", that this interplay between our metric and polarization only holds under specific contexts (i.e. when we have a partition that defines few groups of similar sizes). This assumption is something that we believe is important to verify during the use of our pipeline.

We show what would happen if this assumption would not hold in Figures S10 and S11. We apply the partitioning method we use to the presented networks in the figure, excluding the trivial optimal partition in which one of the subsets is the empty set. We find that the optimal partition consists of one node against the others, and therefore the count of frustrated edges grows linearly for each case. The example given would then result in a one-versus-all faction distribution and therefore would not fulfill our assumption and not be eligible to be studied through our pipeline.

Moreover, in our pipeline, we use two other metrics, namely Cohesiveness and Divisiveness, which are useful to detect pathological cases such as this one, in which we would find an indicator that the edges are not coherent with the partition. These metrics are inspired by previous work (5) but we re-normalize them in order to achieve more informative metrics. To find a more detailed explanation of this renormalization, see the section "The FAULTANA Pipeline" under "Re-normalization and global metrics". In the example we show, our re-normalized metrics indicate that the more nodes we add to the network, the less coherent the links are to the partition (i.e. we find lower values of our metrics). Contrarily, the originally defined metric of Cohesiveness does not change, which supports our decision to use a normalized version.

Table S1. Summary statistics of the datasets used to build signed relation networks. BW1 and BW2 are the two networks obtained from the Birdwatch platform. DS is the network obtained from DerStandard.

	Timespan	Users	Edges / % neg.	Interactions/ % neg.
**BW1**	∼5 months	2,676	25,562 / 28%	32,323 / 28%
**BW2**	∼12 months	10,662	235,493 / 38%	301,041 / 38%
**DS**	8 years	14,827	∼5.56M / 41%	∼76M / 17%

Table S2. Birdwatch Global Results. Summary of the optimal partition results for the two datasets. To evaluate the difference in the use of the methods, for BW1, we show results obtained with both the exact and approximated method. SAI_G confidence intervals are obtained from running 10,000 instances of the null model. Divisiveness and Cohesiveness resample uncertainties are obtained by bootstrapping for 10,000 instances.

	**Method**	K^*	**Ratio Size Groups**	**Ratio Internal/ External**	**% Frus Edges**	**Coh/ Norm. Coh**
**BW1**	Exact	2	65/35	67/33	14%	0.929 / 0.201
**BW1**	Approx	2	71/29	66/34	18%	0.901 / 0.182
**BW2**	Approx.	2	73/27	62/38	25%	0.801 / 0.181
	**Method**	**Coh Bootstrapping Interval**	**Div/ Norm. Div**	**Div Bootstrapping Interval**	SAI_G	SAI_G **95% confidence intervals**
**BW1**	Exact	0.004	0.72 / 0.43	0.01	0.668	0.6642-0.6722
**BW1**	Approx	0.005	0.64 / 0.36	0.01	0.563	0.5579-5686
**BW2**	Approx.	0.002	0.674 / 0.294	0.003	0.475	0.4738-0.4780

Table S3. DerStandard Global Results. Summary of the optimal partition results for the dataset obtained from DerStandard. SAI_G confidence intervals are obtained from running 1,000 instances of the null model. Divisiveness and Cohesiveness resample uncertainties are obtained by bootstrapping for 10,000 instances.

	**Method**	$K*$	**Ratio Size Groups**	**Ratio Internal/ External**	**% Frus Edges**	**Coh/ Norm. Coh**
**DS**	Approx.	2	62/37	67/33	29%	0.7006 / 0.1409
	**Method**	**Coh Bootstrapping Interval**	**Div/ Norm. Div**	**Div Bootstrapping Interval**	SAI_G	SAI_G **95% confidence intervals**
**DS**	Approx.	0.0002	0.7301 / 0.2899	0.0003	0.396	0.3951-0.3961

Table S4. Wordshift keywords of peaks in Alignment and notable events during the BW timeline. The upper table shows the first 10 keywords relevant to the context of each identified peak in Figure 2 (main text). We obtain these words by comparing the text of tagged tweets posted in a period surrounding the peaks with the text in the rest of the dataset. In the second part of the table, we collect events that occur close to the time of detected peaks and that help interpret the keywords above.

Peak	Period Covered	Wordshift Keywords
**1st**	Feb, 7th - Feb, 17th	Trump, Energy, Vote, Impeachment, Trial, Plan, Power, Job, Clear, Start
**2nd**	Mar, 9th - Mar, 19th	Read, Vaccine, Give, Call, Death, Story, Fact, Make, Stop, Covid
**3rd**	Apr, 13th - Apr, 23rd	Police, Black, Kill, Shoot, Murder, Justice, Girl, Cop, Name, Verdict
**4th**	Apr, 28th - May, 8th	Election, Want, Trump, Violation, Get, School, Duck, Pandemic, Go, Big
**5th**	Jun, 22nd - Jul, 2nd	Get, Theory, Crime, Likely, Say, Government, Pay, Right, Voter, Collapse
**Tag**	**Date**	**Event Summary**
A	12th February 2021	Governor Abbott Issues Disaster Declaration in relation to the Storms and Power Crisis in Texas
B	11th March 2021	President Biden to Announce All Americans to be Eligible for Vaccinations by May 1
C	11th April 2021	Killing of Daunte Wright (20 years old) by the police during a traffic stop for an outstanding warrant
D	15th April 2021	Release of a relevant body cam video of the killing of Adam Toledo (13 years old) by a CPD Officer
E	21st April 2021	Killing of Ma'Khia Bryant (16 years old) by a police officer in Columbus, Ohio
F	5th May 2021	Facebook's Oversight Board upholds ban on Trump
G	17th June 2021	Biden-Harris Administration Announces Comprehensive Strategy to Prevent and Respond to Gun Crime

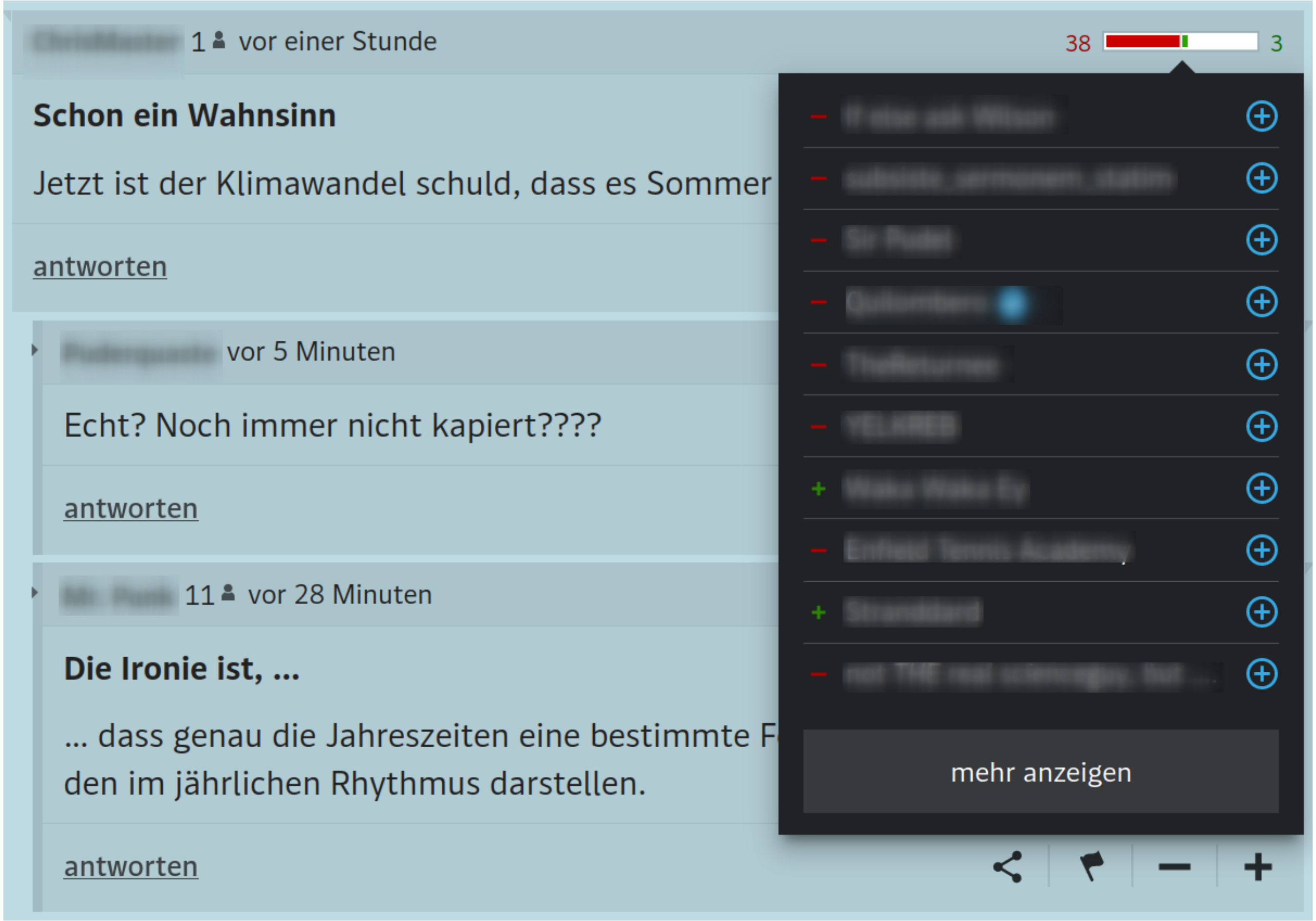

Fig. S1. Example of one DerStandard forum post, showing votes. Each posting in the forum can be up (green) or down votes by other registered user. The bar on the left top of the posting shows the sum for both types of votes. By clicking on that bar, we open a menu that contains the user names of voters and the type of vote cast. (User names have been blurred by the authors in this example.)

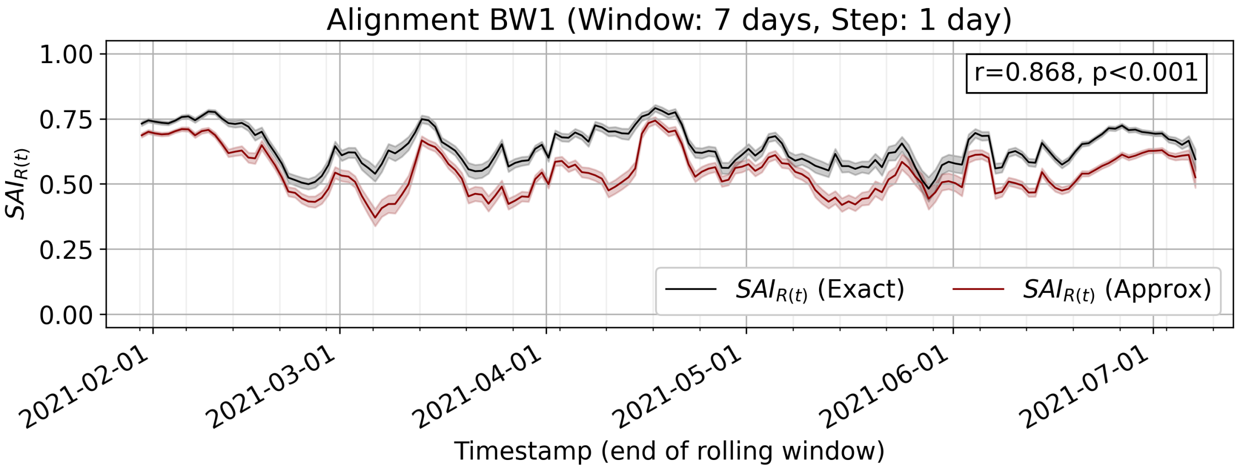

Fig. S2. Comparison between the timeline results obtained for the approximated and exact methods in the BW1 dataset. This figure is an analogous of Figure 4 in the main text with different rolling window parameters. It presents the changes in Alignment obtained with the optimal partition of the exact method and the sub-optimal partition obtained through the approximated algorithm with the same data. Even though the approximated results are consistently lower than the exact results, the variations in the two time series are highly correlated.

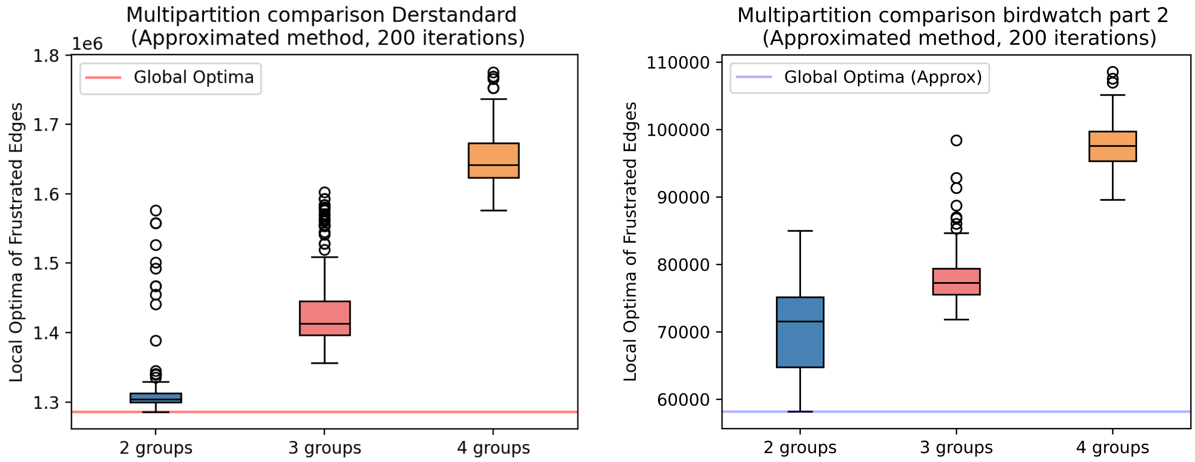

Fig. S3. Multipartition study for Derstandard (left) and BW2 (right). We show the distribution of results for the approximated method for $k = 2, 3$ and 4. In a straight line, we mark the best partition result, which we assume to be the closest to the global optima. All other solutions are sub-optimal and therefore local optima. In both datasets $k = 2$ is the best number of groups.

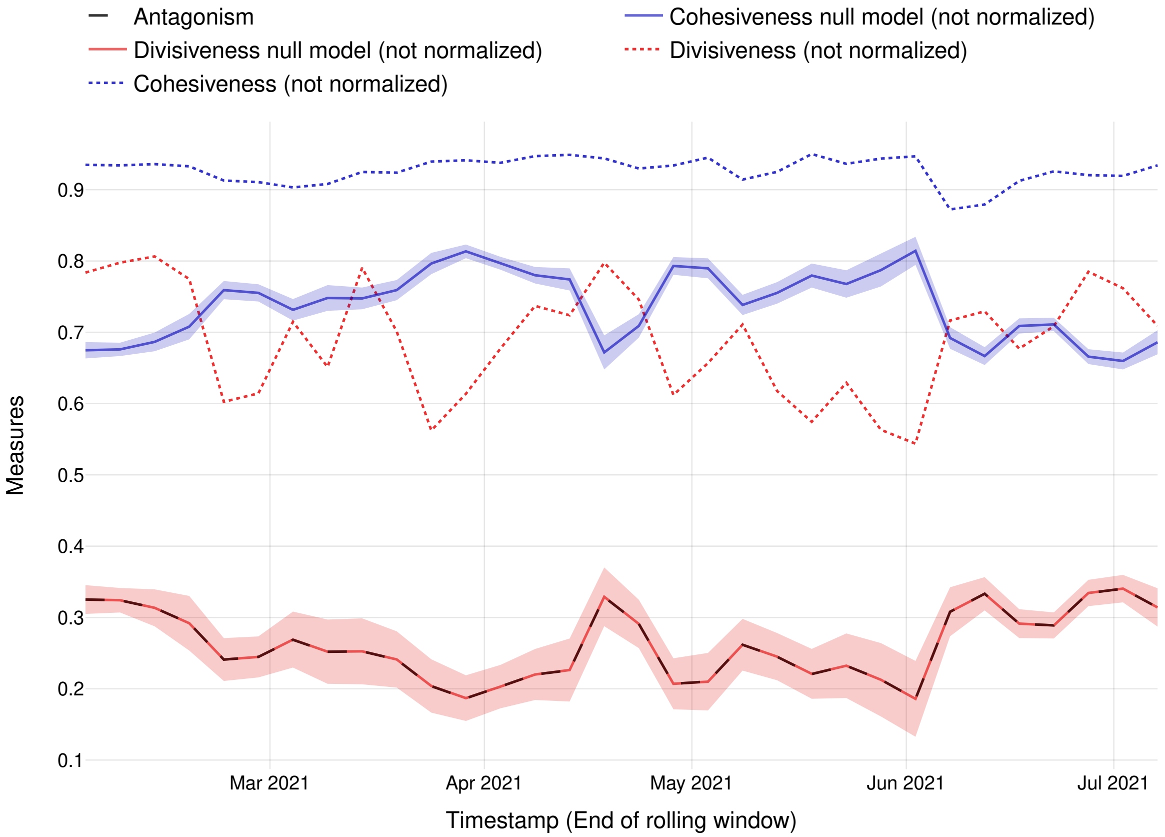

Fig. S4. Cohesiveness and Divisiveness of the original data and null model before normalization for the BW1 time series. We show the metrics of Cohesiveness and Divisiveness for the original data (dotted lines) and for the null model. The null model time series is shown with 95% confidence intervals obtained from the $10,000$ instances of re-shuffled sign distributions. The proportion of negative interactions in each time window is represented in a dashed line and is perfectly correlated to the mean Divisiveness signal of the null model. Note that it is also inversely correlated to the Cohesiveness of the null model.

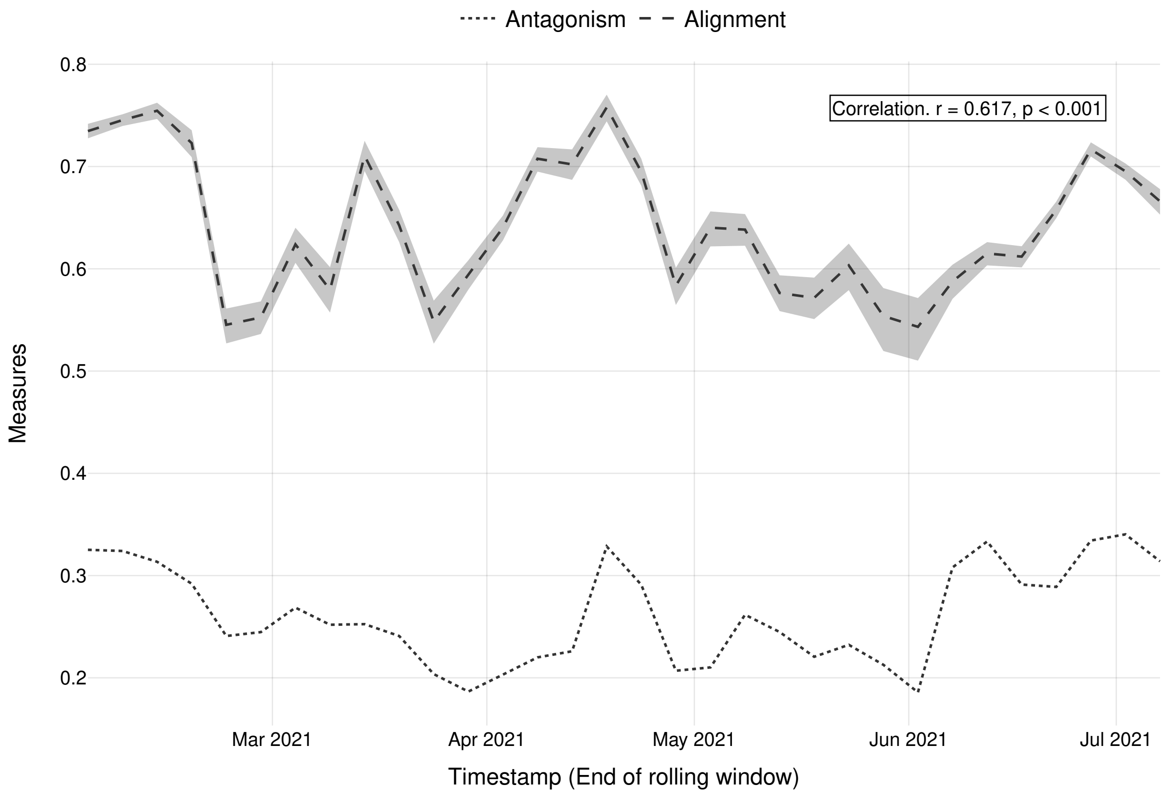

Fig. S5. Antagonism and Alignment of the BW1 time series. We see that, while fluctuations are similar for both metrics in some time windows, the correlation between the metrics is low enough to consider them as separate measures that provide different insights.

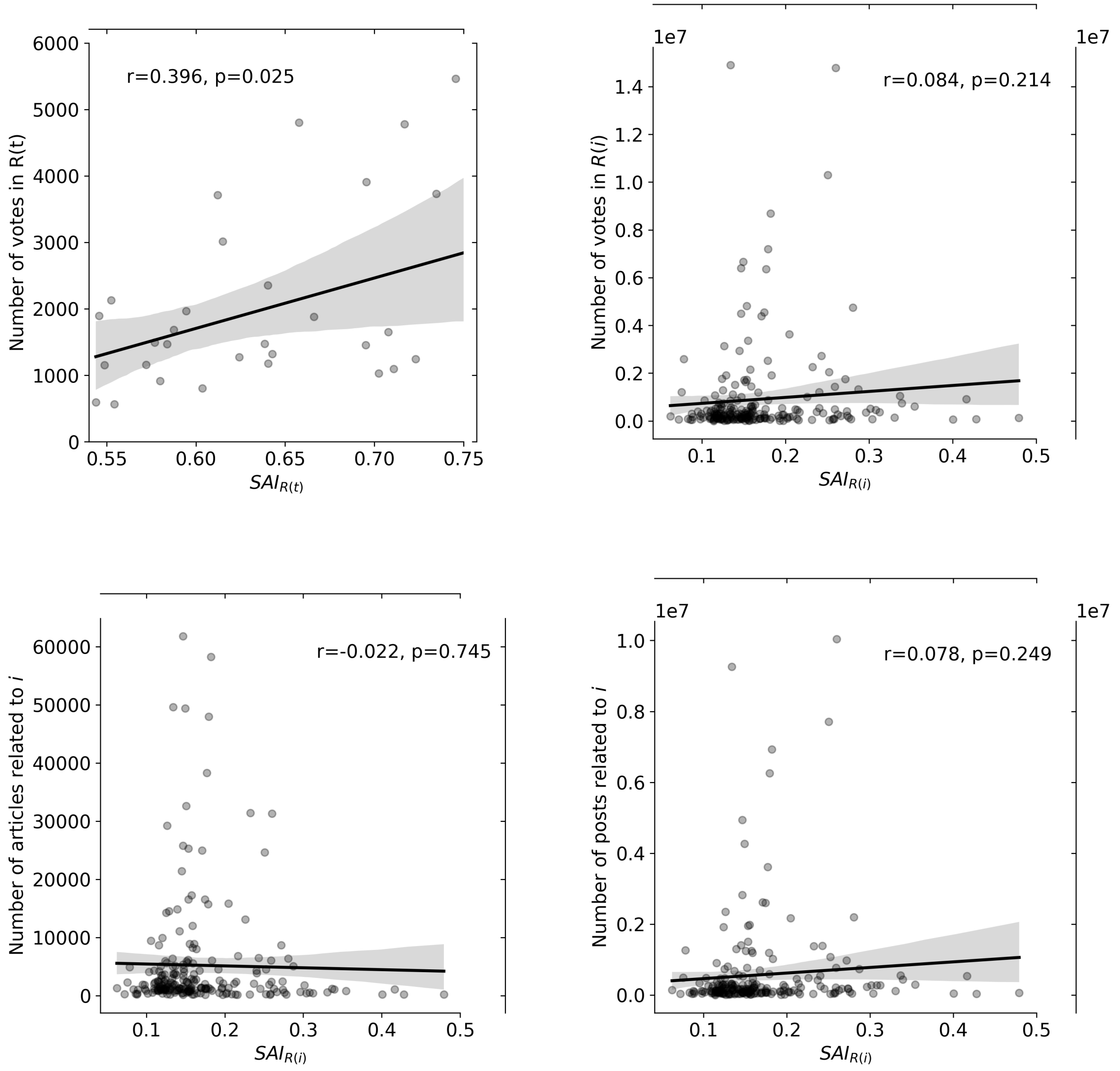

Fig. S6. Correlation between our Alignment metric and the volume of data of studied subsets. Scatter plots showing the correlation of the SAI_R measure against the volume of votes for the timeline of BW1 (top left) and the Antagonism-Alignment study for Derstandard (top right). The two lower figures similarly indicate the correlation between the Alginment measures and the volume of articles and posts obtained for each issue for the Derstandard study.

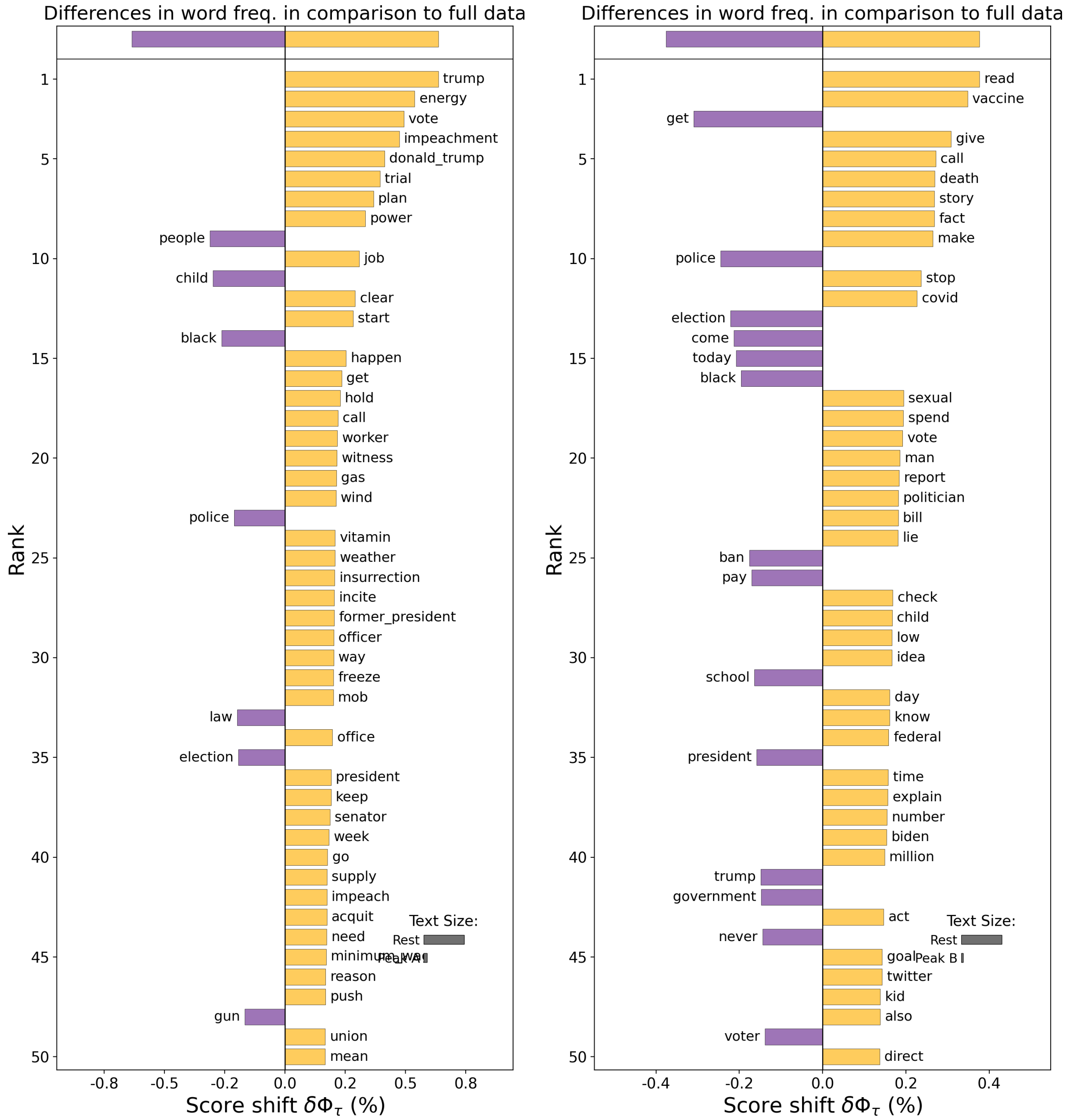

Fig. S7. Wordshift graphs for peak A (left) and peak B (right).

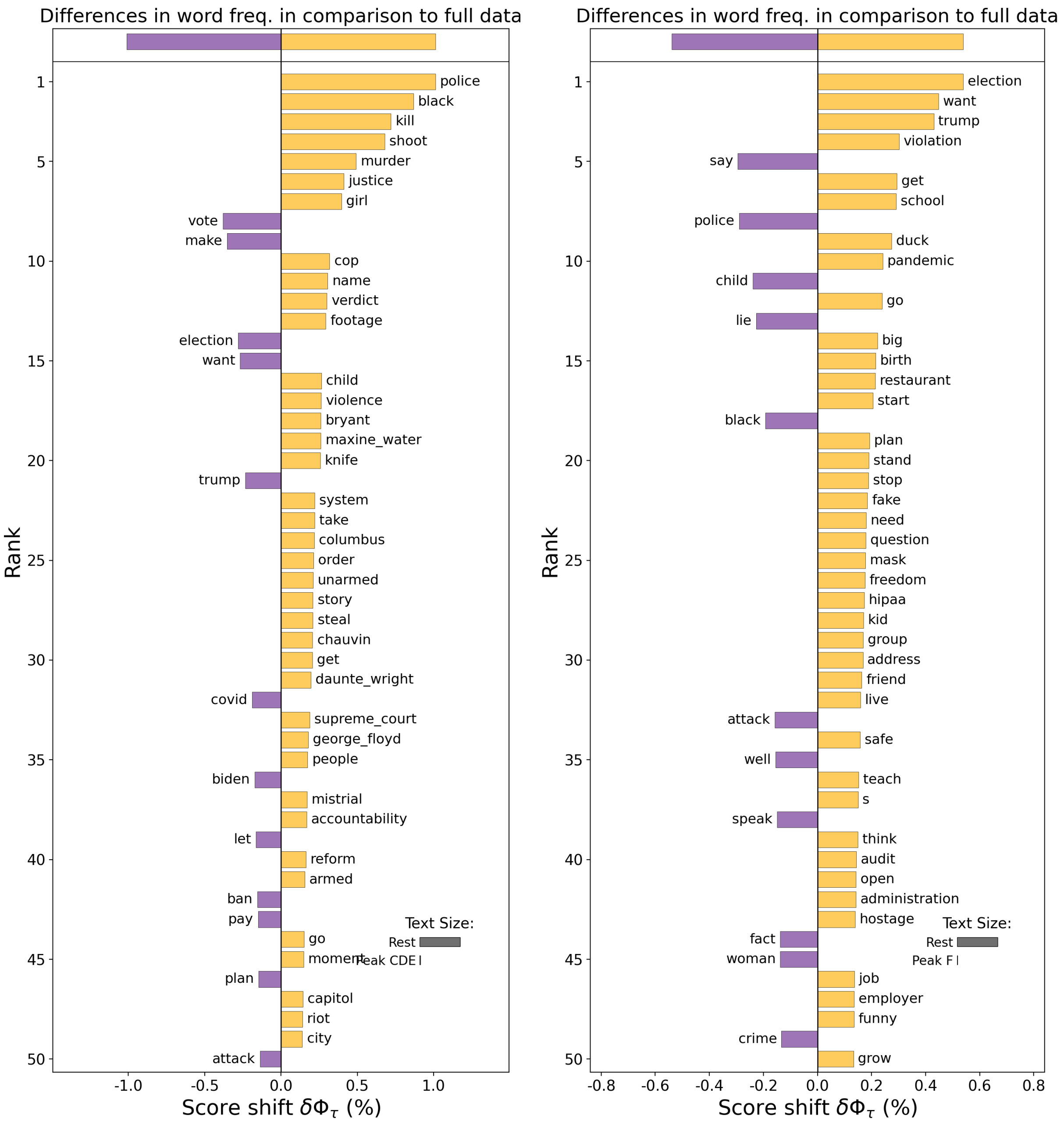

Fig. S8. Wordshift graphs for peak CDE (left) and peak G (right).

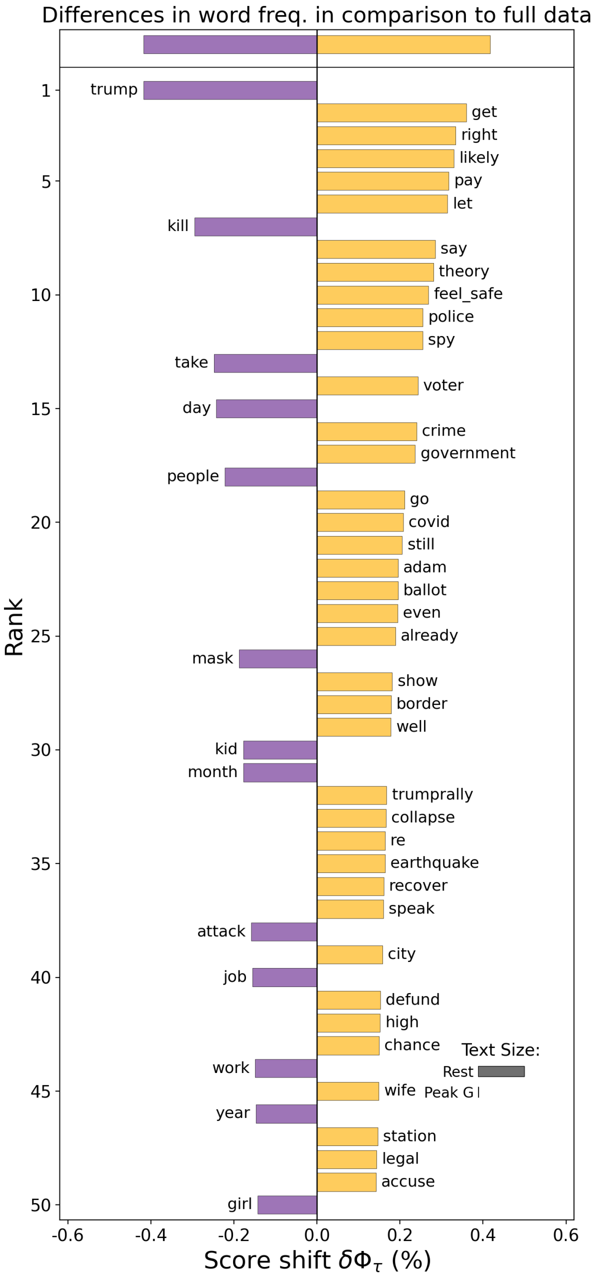

Fig. S9. Wordshift graph for peak F.

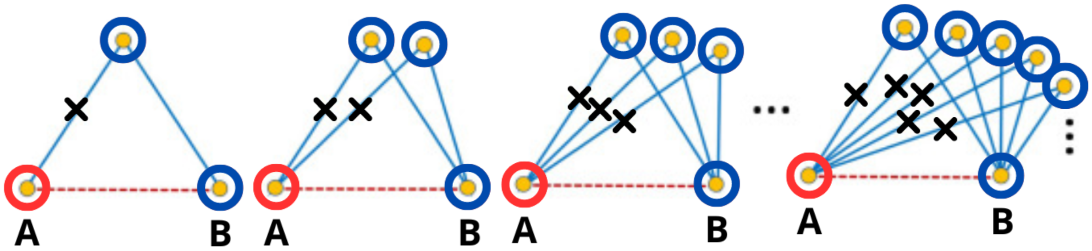

Fig. S10. Problematic example for frustration based assessment of polarization. We show an ill-defined example marked with the optimal partition obtained by minimizing the number of frustrated edges. For each case, there is an alternative optimal partition which would simply correspond to switching the labels of nodes A and B. Frustrated edges are tagged with a cross. The original image of the diagram is extracted from (6). In Fig.S11 we show the resulting metrics of each case in this example.

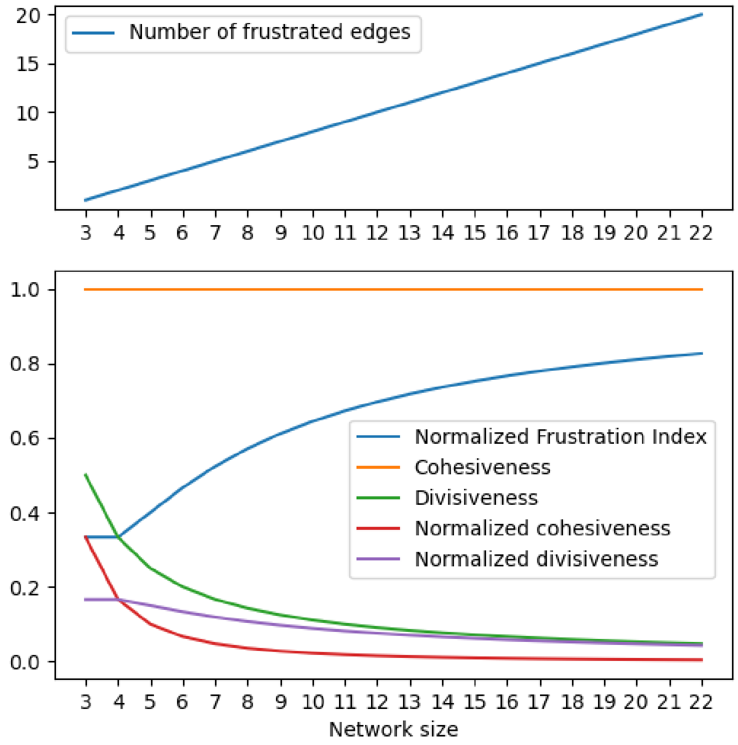

Fig. S11. Metrics obtained from the generalization of the example shown in Fig.S10 when consecutively adding one node with a positive link to node A and a positive link to node B. The number of frustrated edges (upper figure) grows linearly. In the lower figure, we show some related metrics: The normalized Frustration Index (4), which grows with the number of added nodes, except for the first two cases; the metrics of Cohesiveness and Divisiveness, which are defined by the proportion of internal (external) edges that are positive (negative), correspondingly; and our normalized metrics for cohesiveness and divisiveness, which also account for the imbalance between positive and negative links of the network. The normalized metrics show the difference between each case better, and reach considerably low values for cases with more nodes, indicating a not very coherent partition.

References

1. P Doreian, A Mrvar, Partitioning signed social networks. *Soc. Networks* **31**, 1–11 (2009).
2. RJ Gallagher, et al., Generalized word shift graphs: a method for visualizing and explaining pairwise comparisons between texts. *EPJ Data Sci.* **10**, 4 (2021).
3. SC Boulila, C Carri, On cologne: Gender, migration and unacknowledged racisms in germany. *Eur. J. Women's Stud.* **24**, 286–293 (2017).
4. S Aref, MC Wilson, Measuring partial balance in signed networks. *J. Complex Networks* **6**, 566–595 (2018).
5. S Aref, L Dinh, R Rezapour, J Diesner, Multilevel structural evaluation of signed directed social networks based on balance theory. *Sci. reports* **10**, 1–12 (2020).
6. E Estrada, Rethinking structural balance in signed social networks. *Discret. Appl. Math.* **268**, 70–90 (2019).
